# Drought and Nitrogen Deposition Drive Community Restructuring of Endophytic Fungi and Functional Regulation by Dark Septate Endophytes in *Quercus dentata*

**DOI:** 10.3390/jof12050324

**Published:** 2026-04-28

**Authors:** Zipeng Zhao, Xiaona Wang, Yafei Ding, Taian Hou, Xingdian Zhang

**Affiliations:** 1 College of Horticulture and Forestry, Tarim University, Alar 843300, China; hauzzp@163.com (Z.Z.);; 2 College of Landscape Architecture and Tourism, Hebei Agricultural University, Baoding 071000, China; 3 The National and Local Joint Engineering Laboratory of High Efficiency and Superior-Quality Cultivation and Fruit Deep Processing Technology of Characteristic Fruit Trees in Southern Xinjiang, Alar 843300, China; 4 College of Forestry, Hebei Agricultural University, Baoding 071000, China

**Keywords:** culturable endophytic fungi, photosynthesis, nutrient uptake, fungal community structure, plant–microbe interactions

## Abstract

Drought and nitrogen deposition are major drivers of global change that can influence forest ecosystems and plant–microbe interactions, yet their combined effects on endophytic fungal communities and the roles of dark septate endophytes (DSE) remain unclear. In this study, we examined the diversity of culturable endophytic fungi in leaves and roots of *Quercus dentata* under different drought and nitrogen deposition treatments and evaluated the functional effects of representative DSE strains on host growth and physiology. A total of 1488 fungal isolates were obtained, revealing distinct organ-specific community patterns. Root-associated communities showed greater compositional stability across treatments, whereas leaf communities were more responsive to environmental variation. Severe drought reduced the dominance of several genera and promoted community restructuring, while nitrogen deposition had contrasting effects on α-diversity in leaves and roots. Beta diversity analyses indicated significant interaction effects between drought and nitrogen addition. Inoculation with four DSE strains produced strain-dependent effects on plant biomass, photosynthesis, water-use efficiency, physiological traits, and nutrient contents. These results indicate that drought and nitrogen deposition jointly influence endophytic fungal communities and that functional differences among DSE strains may affect host responses to combined stress.

## 1. Introduction

Global climate change is increasingly altering terrestrial ecosystem processes through rising atmospheric nitrogen deposition and intensified drought events [1,2]. Nitrogen deposition, primarily derived from anthropogenic activities such as fossil fuel combustion and agricultural fertilization, has substantially increased over the past decades and is projected to continue rising in many regions [3]. At the same time, climate models predict more frequent and severe drought episodes under future warming scenarios [4,5]. These global change drivers profoundly influence plant growth, nutrient cycling, and belowground microbial communities, often through complex and interactive mechanisms. Understanding how plants and their associated microorganisms respond to combined drought and nitrogen enrichment is therefore critical for predicting forest ecosystem stability under future climate conditions.

Endophytic fungi, which reside asymptomatically within plant tissues, are recognized as key components of plant-associated microbiomes [6,7,8]. They can enhance host tolerance to abiotic stress, improve nutrient acquisition, and modulate physiological processes [9,10,11]. The structure and diversity of endophytic fungal communities are strongly shaped by environmental conditions, host genotype, and tissue type [12,13,14]. Previous studies have demonstrated that drought and nitrogen addition can significantly alter fungal community composition in both leaves and roots; however, responses are often organ-specific and context-dependent [15,16,17]. Moreover, most existing studies have focused on either aboveground or soil fungal communities, while comparatively fewer have simultaneously examined endophytic fungi across plant organs under combined stress conditions.

Among endophytic fungi, dark septate endophytes (DSE) represent a widespread and ecologically important functional group characterized by melanized, septate hyphae and frequent microsclerotia formation [18]. DSE colonize plant roots across diverse ecosystems, particularly in stressful environments, and are increasingly recognized for their potential roles in enhancing host drought tolerance, nutrient uptake, and stress resilience [19,20]. Nevertheless, DSE–plant interactions are highly variable and may range from mutualistic to neutral or even parasitic, depending on fungal strain, host species, and environmental context [21]. Functional differentiation among DSE strains remains insufficiently understood, especially under combined global change drivers such as drought and nitrogen deposition.

*Quercus dentata*, a dominant broad-leaved tree species in temperate forests of Northeast Asia, plays an important ecological role in maintaining forest structure and biodiversity. Given its wide distribution and ecological significance, understanding how its associated fungal communities respond to environmental stress is essential for predicting forest ecosystem responses to climate change [22,23]. However, information on the diversity of culturable endophytic fungi in *Q. dentata* and their functional roles under combined drought and nitrogen deposition remains limited [24].

In this study, we investigated how drought and nitrogen deposition influence the community composition and diversity of culturable endophytic fungi in leaves and roots of *Q. dentata* and how representative DSE strains affect plant growth, photosynthesis, physiological traits, and nutrient acquisition under combined stress. We hypothesized that (1) drought and nitrogen deposition would differentially reshape fungal communities in leaves and roots, with stronger compositional shifts under combined stress, and (2) DSE strains would exhibit distinct functional strategies, leading to variable effects on host performance. By integrating community-level analyses with strain-level functional experiments, this study aims to provide mechanistic insights into plant–microbe interactions under global change scenarios.

## 2. Materials and Methods

### 2.1. Experimental Design for Nitrogen and Drought Treatments

This experiment was conducted under natural light conditions at Hebei Agricultural University (115°45′ E, 38°50′ N). Uniform two-year-old *Quercus dentata* seedlings were selected and transplanted into plastic pots (31 cm in height and 28 cm in diameter) filled with a soil mixture of loam, sand, and peat at a volume ratio of 4:2:1.

A factorial design combining nitrogen addition and drought stress was established. Nitrogen was applied at two levels: 0 kg ha^−1^ year^−1^ (N0, control) and 150 kg ha^−1^ year^−1^ (N150). Soil moisture was maintained at three levels corresponding to 80% (W80, well-watered), 50% (W50, moderate drought), and 20% (W20, severe drought) of soil relative water content (RWC), resulting in six treatment combinations (N0W80, N0W50, N0W20, N150W80, N150W50, and N150W20).

Nitrogen was supplied every 14 days as a urea solution [(NH_2_)_2_CO], dissolved in 0.5 L of distilled water per pot. Control plants received an equal volume of distilled water. Soil RWC was monitored using a portable TDR meter (Field Scout TDR300, Spectrum Technologies, Aurora, IL, USA) and adjusted throughout the growing season (May–October) to maintain target moisture levels.

At the end of the treatment period, leaf and fine root samples (<2 mm in diameter) were collected from randomly selected seedlings within each treatment for subsequent fungal isolation and community analysis.

### 2.2. Isolation and Identification of Culturable Endophytic Fungi

Sample preparation and surface sterilization: Fresh and healthy leaves and fine roots were collected from *Q*. *dentata* seedlings under each treatment. Samples were first rinsed with sterile distilled water to remove surface debris. Surface sterilization was performed by immersion in 75% ethanol for 1 min, followed by 10% sodium hypochlorite for 3 min. After disinfection, samples were rinsed three times with sterile distilled water to remove residual disinfectants. Sterilized tissues were air-dried on sterile filter paper under aseptic conditions. This sterilization procedure is commonly used in studies of endophytic fungi to eliminate epiphytic microorganisms, although such treatments may influence the recovery of some sensitive endophytic taxa [25].

Fungal isolation: Surface-sterilized leaves were cut into approximately 5 mm × 5 mm segments, and fine roots were cut into 5 mm fragments using a sterile scalpel. Four to five segments from each sample were aseptically placed onto potato dextrose agar (PDA) plates supplemented with 50 μg mL^−1^ streptomycin and 50 μg mL^−1^ penicillin to inhibit bacterial growth. Plates were incubated at 28 °C in darkness and monitored daily. Emerging fungal colonies were transferred to fresh PDA plates to obtain pure cultures.

For each treatment, 100 leaf tissue plates and 60 root tissue plates were prepared. Each plate contained five leaf segments or four root segments.

Fungal identification: Fungal isolates were initially grouped based on colony morphology and microscopic characteristics, including colony color, texture, growth rate, pigmentation, hyphal structure, and spore morphology. Representative isolates were selected for molecular identification. Approximately 100 mg of fresh fungal biomass was harvested from actively growing cultures, and genomic DNA was extracted using a commercial fungal DNA extraction kit (Sangon Biotech Co., Ltd., Shanghai, China) according to the manufacturer’s instructions.

Molecular analysis: The internal transcribed spacer (ITS) region of rDNA was amplified using universal primers ITS1 and ITS4 [26]. PCR products were purified and sequenced by a commercial sequencing service. Obtained sequences were compared with those available in the NCBI GenBank database using the BLASTn algorithm for species-level identification.

### 2.3. Phylogenetic Analysis of the Four Selected DSE Strains

ITS sequences of the selected DSE strains, *Chaetomium globosum* (Cg, GenBank: PQ462528), *Acrocalymma vagum* (Av, GenBank: PQ462537), *Paraphoma chrysanthemicola* (Pc, GenBank: PQ462608), and *Tetracladium maxilliforme* (Tm, GenBank: PQ462609), were used for phylogenetic analysis together with related reference sequences retrieved from GenBank. The strains were selected from the pool of successfully isolated DSE taxa; among isolates showing stable growth under laboratory conditions, four strains were randomly chosen for further analyses and reinoculation experiments. Sequence alignment was performed using MEGA 12. A phylogenetic tree was constructed using the maximum likelihood method with 1000 bootstrap replicates. *Talaromyces trachyspermus* CBS 373.48 was used as the outgroup.

### 2.4. Reinoculation Experiment and Experimental Design

Based on the isolation, identification, and phylogenetic analysis described above, the four selected DSE strains were used for reinoculation experiments to evaluate their effects on *Q. dentata* seedlings.

Seed preparation and seedling cultivation: *Q. dentata* seeds were collected from the Hongyashan State-Owned Forest Farm Management Bureau (Baoding, Hebei, China). Seeds were surface-sterilized in 70% ethanol for 3 min, followed by immersion in 2.5% sodium hypochlorite for 10 min, and then rinsed three times with sterile distilled water. This sterilization protocol is commonly applied in seed disinfection to ensure aseptic germination in endophyte inoculation studies [27]. Sterilized seeds were mixed with moist sterile sand and stratified at 4 °C until radicle emergence.

Germinated seeds were transplanted into sterilized soil (garden soil: sand = 2:1, *v*/*v*) that had been autoclaved at 121 °C for 2 h. Seedlings were grown in sterilized planting bags and irrigated with sterile water.

DSE inoculation procedure: Actively growing mycelial plugs (9 mm in diameter) were excised from PDA cultures and used as inoculum. Approximately 20 g of inoculated PDA medium was placed into each pot. Control pots received an equal amount of sterile PDA medium without fungal inoculation.

Uniform one-year-old *Q. dentata* seedlings approximately 10 cm in height were selected. Five seedlings were transplanted into each pot. Each inoculation treatment included four replicates under natural light conditions.

After one month of growth, root colonization was examined microscopically to confirm successful DSE infection. Upon confirmation of colonization, seedlings were subjected to the N150W20 treatment for functional evaluation.

### 2.5. Plant Sampling and Measurement Parameters

After 90 days of treatment, seedlings were carefully removed from the pots and gently rinsed with distilled water to remove adhering soil particles. Each treatment included four biological replicates (*n* = 4). Plants were first heated at 105°C for 20 min to inactivate enzymatic activity and then oven-dried at 65°C to constant weight. Dry biomass was determined using an analytical balance. Dried leaf samples were subsequently used for nutrient element analysis. Fresh leaf tissues designated for physiological measurements were immediately frozen in liquid nitrogen and stored at −80°C until further analysis.

#### 2.5.1. Gas Exchange Parameters

Leaf gas exchange parameters were measured using a LI-6400XT portable photosynthesis system (LI-COR Inc., Lincoln, NE, USA). The recorded parameters included net photosynthetic rate (*P*_n_), stomatal conductance (*G*_s_), intercellular CO_2_ concentration (*C*_i_), and transpiration rate (*T*_r_). Measurements were conducted between 8:30 and 10:30 a.m. on clear days. During measurements, photosynthetically active radiation (PAR) was maintained at a constant level of 900 μmol m^−2^ s^−1^ using a built-in LED light source. Leaves were carefully positioned to fully cover the chamber window while avoiding major veins.

Water-use efficiency (WUE) was calculated as the ratio of *P*_n_ to *T*_r_.

#### 2.5.2. Physiological Characteristics

Superoxide dismutase (SOD) activity was evaluated using the nitroblue tetrazolium (NBT) method [28]. Catalase (CAT) activity was determined using the potassium permanganate titration method [29]. Malondialdehyde (MDA) content was determined using the thiobarbituric acid method [30]. Free proline content was measured using the acid ninhydrin method [31]. Soluble sugar (SS) content was determined using the phenol-sulfuric acid method [32]. Soluble protein (SP) content was measured according to the Coomassie brilliant blue method [33].

#### 2.5.3. Nutrient Element Analysis

Dried leaf samples were finely ground into a homogeneous powder. A known mass of each sample was digested using a mixture of concentrated sulfuric acid (H_2_SO_4_) and hydrogen peroxide (H_2_O_2_), following the method described by Buondonno [34].

Total nitrogen (N) content was determined using an automated chemical analyzer. Phosphorus (P) content was measured using the molybdenum–antimony colorimetric method. Potassium (K), calcium (Ca), magnesium (Mg), boron (B), and zinc (Zn) concentrations were quantified using inductively coupled plasma optical emission spectrometry (ICP-OES; Prodigy, Leeman Labs Inc., Hudson, NH, USA).

### 2.6. Statistical Analyses

All statistical analyses were performed using R software (version 4.3.1). Alpha diversity indices, including Shannon, Simpson, Pielou’s evenness, and Chao1 richness, were calculated. Beta diversity was assessed based on Bray–Curtis dissimilarity matrices and visualized using non-metric multidimensional scaling (NMDS) ordination. Differences in community composition among treatments were further tested using permutational multivariate analysis of variance (PERMANOVA). Differences among treatments were analyzed using one-way analysis of variance (ANOVA). When significant differences were detected, Duncan’s multiple range test was applied for post hoc comparisons. Statistical significance was determined at *p* < 0.05.

## 3. Results

### 3.1. Culturable Endophytic Fungi Community Composition in Leaves and Roots

A total of 1488 culturable fungal isolates were obtained from *Q*. *dentata* leaves and roots across the six drought and nitrogen deposition treatments. The culturable endophytic fungi isolated from leaves were assigned to 2 phyla, 10 classes, and 48 genera, whereas those from roots belonged to 2 phyla, 8 classes, and 51 genera.

**Figure 1:**
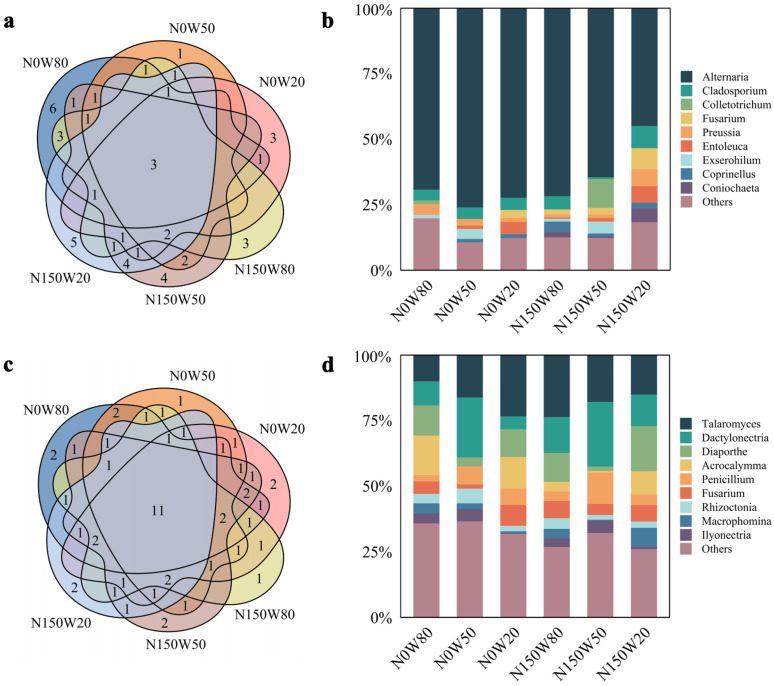
Venn diagrams and relative abundance charts showcasing the diversity and distribution of endophytic fungi within *Quercus dentata* leaves (**a**,**b**) and roots (**c**,**d**) under drought and nitrogen deposition treatments. (**a**) and (**c**) Venn diagrams depict the unique and shared genera of endophytic fungi in leaves and roots, respectively, under different treatments. The numbers in the Venn diagrams represent the number of fungal genera identified. (**b**) and (**d**) display the relative abundance of the top endophytic fungal genera in leaves and roots, respectively, under the same set of treatments. N0 and N150 denote 0 and 150 kg N ha^−1^ yr^−1^ nitrogen addition, respectively, while W80, W50, and W20 represent 80%, 50%, and 20% soil water content, respectively.

Venn diagrams revealed clear differences in fungal genera among treatments in leaves and roots (Figure 1a,c). In leaves, 18, 11, and 12 genera were detected under N0W80, N0W50, and N0W20, respectively, whereas 19, 24, and 19 genera were recorded under N150W80, N150W50, and N150W20 (Figure 1a). *Alternaria*, *Cladosporium*, and *Preussia* were shared among all leaf treatments. Several genera were treatment-specific. N0W80 showed the highest number of unique genera, with six genera detected only in this treatment, including *Acrocalymma*, *Amycosphaerella*, *Coniothyrium*, *Erythrobasidium*, *Petriella*, and *Rosellinia*. In contrast, only one unique genus, *Cercospora*, was found under N0W50. Three genera, *Arthrinium*, *Didymella*, and *Sympodiomycopsis*, were unique to N0W20. Under nitrogen addition, N150W80 contained three unique genera: *Chordomyces*, *Moesziomyces*, and *Sarocladium*. N150W50 was characterized by four unique genera, namely *Diaporthe*, *Penicillium*, *Periconia*, and *Plectosphaerella*. Five unique genera were detected in N150W20, namely *Acremonium*, *Daldinia*, *Dichotomopilus*, *Nemania*, and *Sphaerosporella*.

In roots, 30 genera were detected under both N0W80 and N0W50, whereas 34 genera were recorded under N0W20 (Figure 1c). Under nitrogen addition, 27, 30, and 24 genera were detected under N150W80, N150W50, and N150W20, respectively. Eleven genera—*Aspergillus*, *Dactylonectria*, *Diaporthe*, *Fusarium*, *Harknessia*, *Ilyonectria*, *Macrophomina*, *Penicillium*, *Phialophora*, *Rhizoctonia*, and *Talaromyces*—were shared across all root treatments. Several genera were treatment-specific. *Myrmecridium* and *Phoma* were detected only under N0W80, whereas *Oidiodendron* was unique to N0W50. Two genera, *Thelonectria* and *Cistella*, were found only under N0W20. Under nitrogen addition, *Fusidium* was unique to N150W80, while *Coniothyrium* and *Nigrospora* were unique to N150W50. In N150W20, the unique genera were *Meyerozyma* and *Phialemonium*.

The relative abundance of culturable endophytic fungi differed markedly between leaves and roots (Figure 1b,d). In leaves, Alternaria was dominant across all treatments and accounted for 69.18% of the total abundance under N0W80 (Figure 1b). Under W20, the relative abundance of *Alternaria* declined, whereas *Entoleuca* increased. Under N150W50, the relative abundance of *Colletotrichum* increased, accompanied by a reduction in *Alternaria*. Under severe drought combined with nitrogen addition, the relative abundances of *Entoleuca*, *Fusarium*, *Coniochaeta*, and *Cladosporium* increased, and the proportion of *Alternaria* decreased to 44.95%.

In contrast, the fungal community structure in roots differed substantially from that in leaves (Figure 1d). Although *Alternaria* remained relatively abundant, it was no longer the dominant genus under some treatments. Under N0W80, *Acrocalymma* was the most abundant genus, accounting for 15.16% of the total abundance. Under W50, regardless of nitrogen addition, *Dactylonectria* became dominant and accounted for 22.87% and 24.71% of the community under N0W50 and N150W50, respectively. Under N150W20, the relative abundance of *Diaporthe* increased to 17.12%, making it the dominant genus. Overall, drought and nitrogen addition altered the dominant genera in roots more markedly than in leaves.

### 3.2. Alpha and Beta Diversity of Culturable Endophytic Fungi in Leaves and Roots

**Figure 2:**
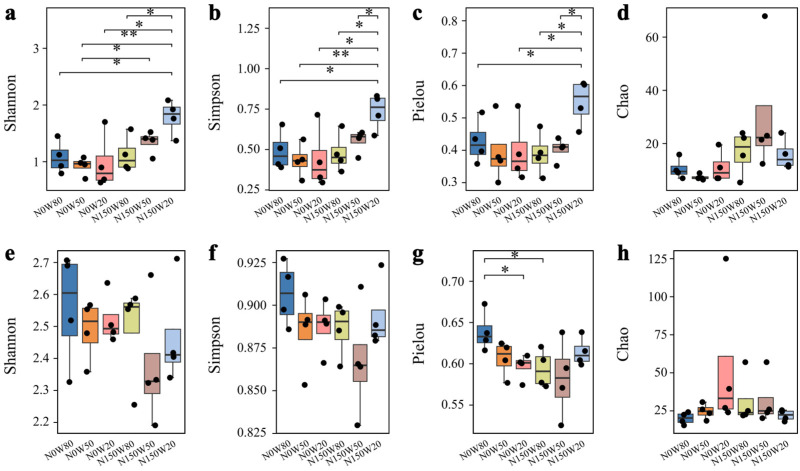
Analysis of alpha diversity indices of endophytic fungi in *Quercus dentata* leaves and roots under drought and nitrogen deposition treatments. Alpha diversity was assessed using the Shannon index (**a**,**e**), Simpson index (**b**,**f**), Pielou index (**c**,**g**), and Chao index (**d**,**h**), reflecting the diversity, evenness, and richness of endophytic fungal communities in leaves (**a**–**d**) and roots (**e**–**h**). N0 and N150 denote 0 and 150 kg N ha^−1^ yr^−1^ nitrogen addition, respectively, while W80, W50, and W20 represent 80%, 50%, and 20% soil water content, respectively. Boxes represent the interquartile range, the horizontal line within each box indicates the median, whiskers indicate the data range, and black dots represent individual replicates. Significant differences among treatments are indicated by asterisks (* *p* < 0.05, ** *p* < 0.01).

Differences in alpha diversity were observed among drought and nitrogen deposition treatments in both leaves and roots (Figure 2). In leaves, the Shannon index was higher under N150W50 and N150W20 than under the other treatments. The Simpson index reached its highest value under N150W20, although the differences among the other treatments were not significant. A similar pattern was found for the Pielou index, which was also higher under N150W50 and N150W20. By contrast, the Chao index did not vary significantly among treatments.

In roots, alpha diversity showed a different response pattern (Figure 2e–h). The Shannon index was highest under N0W80 and tended to decline with decreasing soil moisture and nitrogen addition (Figure 2e). The Simpson index varied little among treatments, although slightly higher values were observed under N0W80 (Figure 2f). The Pielou index was also highest under N0W80, whereas the Chao index remained relatively stable across treatments (Figure 2g,h).

**Figure 3:**
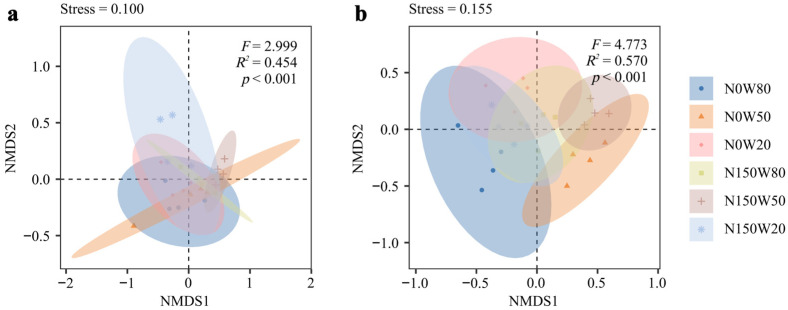
Non-metric multidimensional scaling (NMDS) analysis of endophytic fungal communities in *Quercus dentata* under drought and nitrogen deposition treatments. (**a**) leaves and (**b**) roots. The NMDS plots show the dissimilarity in fungal community composition among different treatments. Each point represents an individual replicate, and the ellipses indicate the 95% confidence intervals for each treatment group. N0 and N150 denote 0 and 150 kg N ha^−1^ yr^−1^ nitrogen addition, respectively, whereas W80, W50, and W20 represent 80%, 50%, and 20% soil water content, respectively.

NMDS ordination further indicated that fungal community composition varied among drought and nitrogen deposition treatments in both leaves and roots (Figure 3). The stress values were 0.100 for leaves and 0.155 for roots, indicating a good representation of the data in two-dimensional space. PERMANOVA analysis further confirmed significant differences in community composition among treatments for both leaves (*F* = 2.999, *R*^2^ = 0.454, *p* < 0.001) and roots (*F* = 4.773, *R*^2^ = 0.570, *p* < 0.001). In leaves, samples under N150W20 were clearly separated from the other treatments. In roots, N0W80 and N150W50 formed distinct groups relative to the remaining treatments.

### 3.3. Phylogenetic Identification, Morphological Characteristics, and Root Colonization of Selected DSE Strains

**Figure 4:**
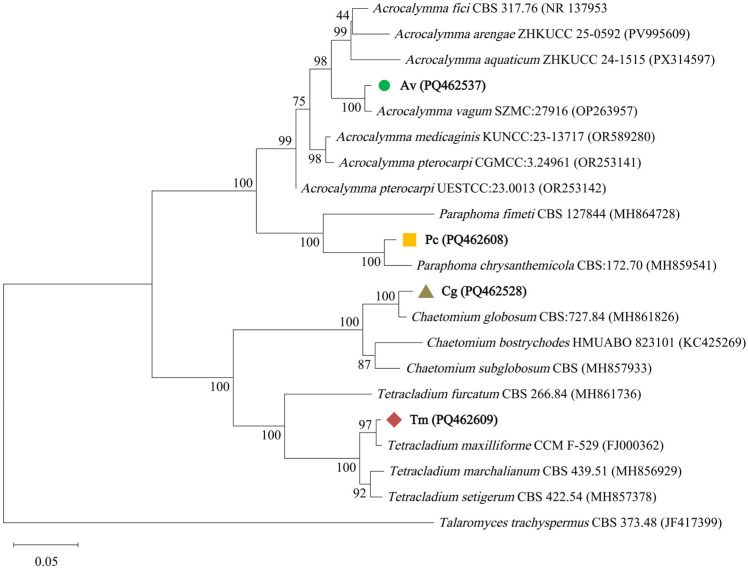
Maximum likelihood phylogenetic tree of endophytic fungal isolates used for reinoculation experiments in *Quercus dentata* based on ITS sequence data. The tree shows the phylogenetic relationships of isolates Av, Pc, Cg, and Tm with related reference taxa. Newly generated isolates in this study are indicated by colored symbols. Bootstrap values from 1000 replicates are shown at the nodes. *Talaromyces trachyspermus* CBS 373.48 was used as the outgroup. The scale bar represents 0.05 substitutions per site.

ITS-based phylogenetic analysis confirmed the identities of the four selected DSE strains used for reinoculation experiments (Figure 4). Strain Cg clustered with *Chaetomium globosum*, Av with *Acrocalymma vagum*, Pc with *Paraphoma chrysanthemicola*, and Tm with *Tetracladium maxilliforme* in the maximum likelihood tree. These results supported the taxonomic identification of the four DSE strains.

**Figure 5:**
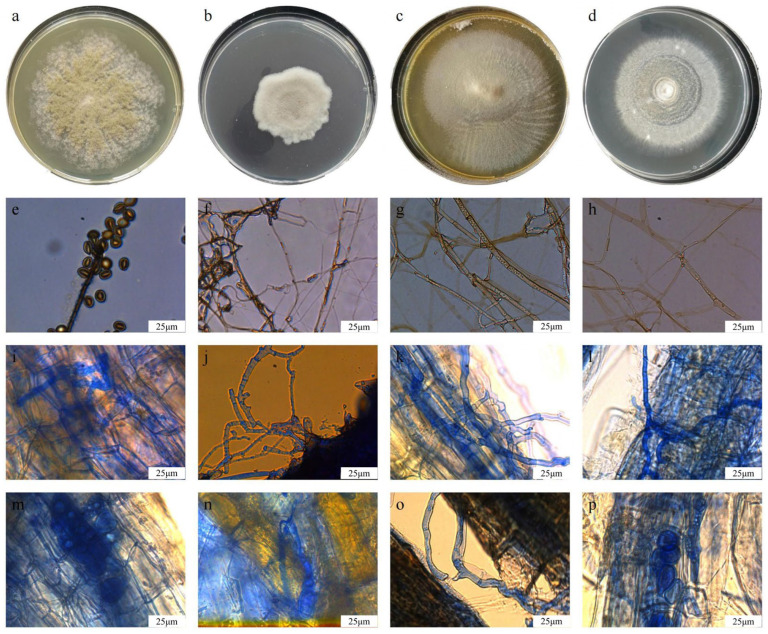
Colony morphology and root colonization characteristics of endophytic fungal isolates after reinoculation into *Quercus dentata* under the N150W20 treatment. (**a**–**d**) Colony morphology of *Chaetomium globosum* (Cg), *Acrocalymma vagum* (Av), *Paraphoma chrysanthemicola* (Pc), and *Tetracladium maxilliforme* (Tm) grown on PDA medium. (**e**–**h**) Hyphal morphology of Cg, Av, Pc, and Tm on PDA medium. (**i**–**l**) Hyphal colonization of Cg, Av, Pc, and Tm in *Q. dentata* roots after reinoculation. (**m**–**p**) Microsclerotia formation of Cg, Av, Pc, and Tm in *Q. dentata* roots after reinoculation. Scale bars = 25 μm.

The four DSE strains differed clearly in colony morphology on PDA medium (Figure 5a–d). Cg formed dense, cottony colonies with a yellowish pigmentation. Av produced compact gray–white colonies with smooth margins. Pc showed radial growth and a light brown colony surface, whereas Tm developed concentric rings with abundant aerial hyphae. Microscopic examination further showed that all four strains possessed typical septate and melanized hyphae (Figure 5e–h). Conidia and microsclerotia were also observed in some isolates, consistent with the morphological characteristics of dark septate endophytes.

After inoculation, all four DSE strains successfully colonized the roots of *Q. dentata* (Figure 5i–p). Melanized hyphae were observed both between and within root cells. Colonization rates varied significantly among strains (Table 1). Tm showed the highest colonization rate at 82.5%, followed by Cg at 67.5% and Pc at 57.5%, whereas Av had the lowest rate at 40.0%. Overall, these observations indicate that all four strains were able to establish stable colonization in *Q. dentata* roots.

### 3.4. Effects of DSE Inoculation on Plant Growth in Quercus Dentata

**Figure 6:**
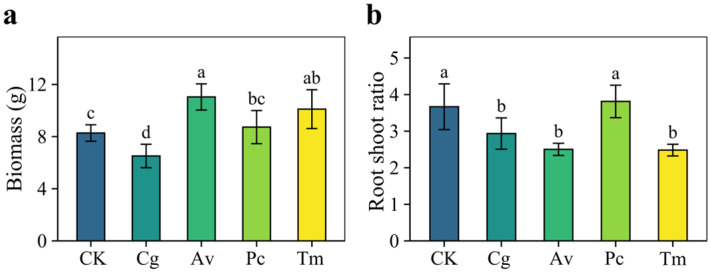
Effects of DSE inoculation on the growth of *Quercus dentata* seedlings under the N150W20 treatment. (**a**) Biomass and (**b**) Root-to-shoot ratio. CK represents the non-inoculated control treatment. Cg, Av, Pc, and Tm represent inoculation with *Chaetomium globosum*, *Acrocalymma vagum*, *Paraphoma chrysanthemicola*, and *Tetracladium maxilliforme*, respectively. Values are presented as mean ± SD (*n* = 4). Different lowercase letters indicate significant differences among treatments at *p* < 0.05 according to Duncan’s test.

Seedling biomass and root-to-shoot ratio varied among DSE inoculation treatments (Figure 6). Inoculation with Av and Tm significantly increased biomass compared with the CK, whereas Pc had no significant effect, and Cg significantly reduced biomass. Among the four strains, Av showed the strongest growth-promoting effect, increasing biomass by 33.47%. The root-to-shoot ratio was significantly reduced by Cg, Av, and Tm, while no significant change was observed in the Pc treatment. The greatest reduction occurred in the Tm treatment, where the root-to-shoot ratio decreased by 32.34%.

### 3.5. Effects of DSE Inoculation on Photosynthesis in Quercus dentata

**Figure 7:**
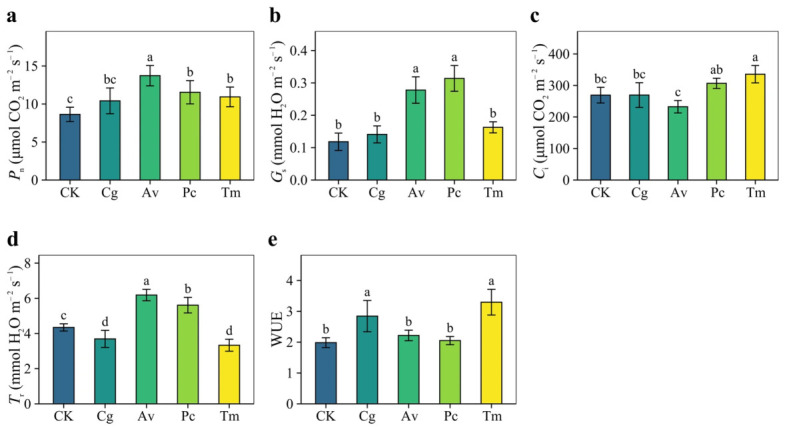
Effects of DSE inoculation on leaf gas exchange parameters of *Quercus dentata* seedlings under the N150W20 treatment. (**a**) Net photosynthetic rate (*P*_n_), (**b**) Stomatal conductance (*G*_s_), (**c**) Intercellular CO_2_ concentration (*C*_i_), (**d**) Transpiration rate (*T*_r_), and (**e**) Water-use efficiency (WUE). CK represents the non-inoculated control treatment. Cg, Av, Pc, and Tm represent inoculation with *Chaetomium globosum*, *Acrocalymma vagum*, *Paraphoma chrysanthemicola*, and *Tetracladium maxilliforme*, respectively. Values are presented as mean ± SD (*n* = 4). Different lowercase letters indicate significant differences among treatments at *p* < 0.05 according to Duncan’s test.

DSE inoculation altered the gas exchange parameters of *Q. dentata* seedlings under combined nitrogen deposition and drought conditions (Figure 7). Overall, all four DSE strains increased *P*_n_ relative to the CK treatment (Figure 7a). The increases were significant in the Av, Pc, and Tm treatments, ranging from 26.64% to 58.89%, whereas the increase in the Cg treatment was comparatively small. *G*_s_ was markedly enhanced by Av and Pc, which increased this parameter by 135% and 165%, respectively (Figure 7b). *C*_i_ increased significantly only in the Tm treatment, by 24.70% (Figure 7c). *T*_r_ showed a strain-dependent response (Figure 7d). It decreased significantly in the Cg and Tm treatments but increased significantly in the Av and Pc treatments compared with CK. WUE was significantly higher in the Cg and Tm treatments, with increases of 43.39% and 66.14%, respectively (Figure 7e).

### 3.6. Effects of DSE Inoculation on Physiological Characteristics in Quercus dentata

**Figure 8:**
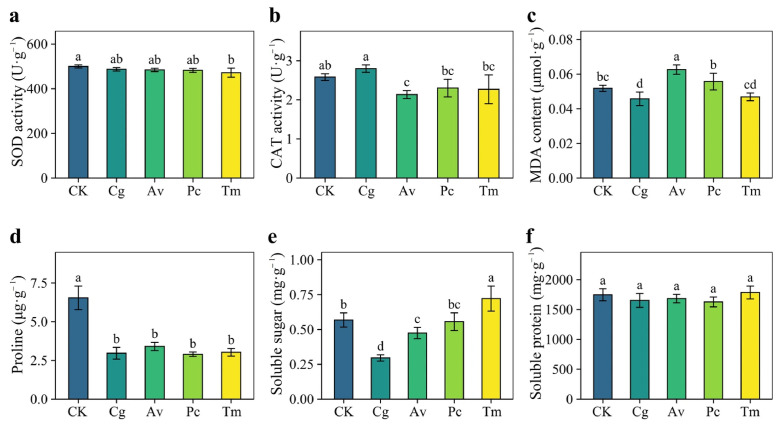
Effects of DSE inoculation on physiological characteristics of *Quercus dentata* seedlings under the N150W20 treatment. (**a**) Superoxide dismutase (SOD) activity, (**b**) Catalase (CAT) activity, (**c**) Malondialdehyde (MDA) content, (**d**) Proline (Pro) content, (**e**) Soluble sugar (SS) content, and (**f**) Soluble protein (SP) content. CK represents the non-inoculated control treatment. Cg, Av, Pc, and Tm represent inoculation with *Chaetomium globosum*, *Acrocalymma vagum*, *Paraphoma chrysanthemicola*, and *Tetracladium maxilliforme*, respectively. Values are presented as mean ± SD (*n* = 4). Different lowercase letters indicate significant differences among treatments at *p* < 0.05 according to Duncan’s test.

Inoculation with DSE strains had no significant effects on SOD activity or soluble protein content (Figure 8a,f). In contrast, CAT activity was significantly reduced by Av inoculation (Figure 8b). MDA content decreased significantly in the Cg treatment but increased significantly in the Av treatment (Figure 8c). Proline content declined markedly in all inoculated treatments, with reductions of more than 48% compared with the CK (Figure 8d). Soluble sugar content was significantly lower under Cg and Av inoculation, whereas it increased significantly in the Tm treatment (Figure 8e).

### 3.7. Effects of DSE Inoculation on Nutrient Contents

**Figure 9:**
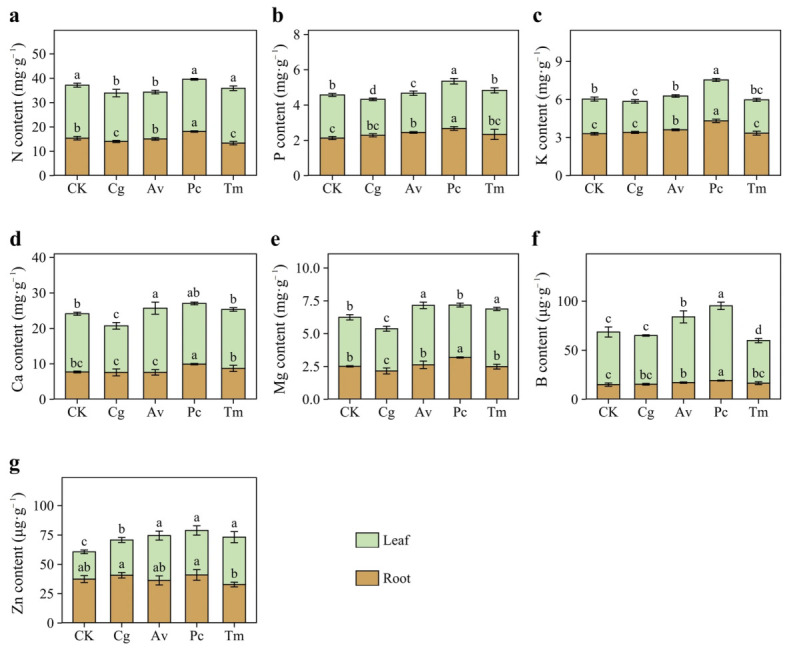
Effects of DSE inoculation on nutrient contents in leaves and roots of *Quercus dentata* seedlings under the N150W20 treatment. (**a**) Nitrogen (N) content, (**b**) Phosphorus (P) content, (**c**) Potassium (K) content, (**d**) Calcium (Ca) content, (**e**) Magnesium (Mg) content, (**f**) Boron (B) content, and (**g**) Zinc (Zn) content. CK represents the non-inoculated control treatment. Cg, Av, Pc, and Tm represent inoculation with *Chaetomium globosum*, *Acrocalymma vagum*, *Paraphoma chrysanthemicola*, and *Tetracladium maxilliforme*, respectively. Values are presented as mean ± SD (*n* = 4). Different lowercase letters indicate significant differences among treatments at *p* < 0.05 according to Duncan’s test.

DSE inoculation affected several nutrient contents in both leaves and roots of *Q. dentata* seedlings, with clear treatment- and organ-specific responses (Figure 9). Leaf N content decreased significantly under Cg and Av inoculation, whereas root N content increased under Pc but decreased under Cg and Tm (Figure 9a). Phosphorus responded most strongly to Pc, with leaf and root P contents increasing by 9.56% and 25.45%, respectively (Figure 9b). Av reduced leaf P content but significantly increased root P content. Cg decreased leaf P content only, while Tm had no significant effect on P content.

Pc also showed the strongest promoting effect on K content, increasing leaf and root K contents by 18.09% and 30.66%, respectively (Figure 9c). Av significantly increased root K content, whereas Cg reduced leaf K content. Ca content increased in leaves under Av inoculation and in roots under Pc and Tm inoculation, while Cg reduced leaf Ca content (Figure 9d). In leaves, Mg content increased under Av and Tm but decreased under Cg. In roots, Mg content increased under Pc but decreased under Cg (Figure 9e).

B content was markedly enhanced by Pc, increasing by 42.03% in leaves and 27.66% in roots (Figure 9f). Av also increased B content in both organs, whereas Tm reduced leaf B content. Leaf Zn content increased significantly in all DSE inoculation treatments, with increases ranging from 29.28% to 73.77% (Figure 9g). Leaf Zn content was significantly higher under Av, Pc, and Tm than under Cg. Root Zn content did not differ significantly from the CK overall, although Pc resulted in a significantly higher value than Tm.

### 3.8. Integrated Clustering Analysis of DSE Functional Traits

**Figure 10:**
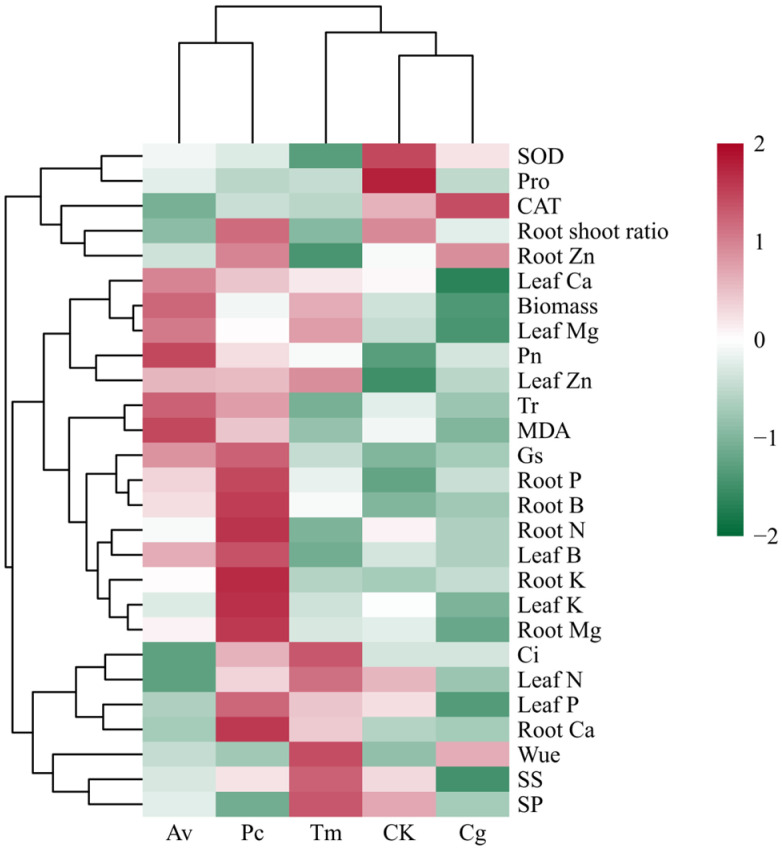
Hierarchical clustering heatmap of standardized functional traits in *Quercus dentata* seedlings following DSE inoculation under the N150W20 treatment. Rows represent measured traits, and columns represent inoculation treatments. Data were standardized using Z-score transformation prior to hierarchical clustering. Red and green indicate relatively higher and lower values, respectively, compared with the overall mean. CK represents the non-inoculated control treatment. Cg, Av, Pc, and Tm represent inoculation with *Chaetomium globosum*, *Acrocalymma vagum*, *Paraphoma chrysanthemicola*, and *Tetracladium maxilliforme*, respectively.

Hierarchical clustering based on standardized functional traits revealed clear differentiation among DSE strains (Figure 10). Av and Pc clustered together, indicating highly similar functional responses characterized by enhanced biomass, photosynthetic performance, and nutrient acquisition. In contrast, Tm formed a distinct cluster associated primarily with increased water-use efficiency and soluble sugar content. Cg grouped closely with the CK, suggesting comparatively weaker functional effects.

Trait clustering further showed that growth, photosynthetic, and nutrient-related variables formed a coordinated module, whereas stress-related indicators such as proline and MDA were grouped separately, demonstrating an inverse relationship with growth-promoting traits.

## 4. Discussion

### 4.1. Effects of Drought and Nitrogen Deposition on Endophytic Fungal Community Structure and Diversity

Drought and nitrogen deposition jointly reshaped the community structure of culturable endophytic fungi in *Q*. *dentata*, with pronounced organ-specific patterns [12]. Leaf and root fungal communities differed substantially in taxonomic composition and dominance structure, reflecting distinct ecological filtering processes operating above- and belowground [20]. Organ-specific differentiation of endophytic assemblages has been widely reported and is often attributed to contrasting microenvironmental conditions and carbon allocation strategies between leaves and roots [7,35]. The relatively higher proportion of shared genera among root treatments in the present study suggests greater stability of root-associated communities across environmental gradients, consistent with the buffering capacity of soil environments and the continuous carbon supply from root exudates [36,37]. In contrast, leaf communities exhibited higher turnover, which agrees with previous findings that phyllosphere fungal assemblages are highly responsive to climatic fluctuations and moisture availability [38,39].

Drought intensity emerged as a major driver of community restructuring. The decline in the dominance of *Alternaria* under severe drought in leaves suggests that environmental stress weakened competitive exclusion and facilitated niche expansion of subordinate taxa [40]. Similar drought-induced reductions in dominant taxa and increased compositional turnover have been reported in forest fungal communities [41]. In roots, the shift in dominant genera along the moisture gradient further supports the idea that water availability acts as a primary environmental filter shaping fungal assemblages [42]. Drought can alter host carbon assimilation, root exudation profiles, and osmotic conditions, thereby modifying fungal colonization success and competitive hierarchies [43]. These patterns are consistent with the stress-gradient framework, which predicts that increasing environmental stress can reduce dominance strength and promote coexistence among taxa [44].

Nitrogen addition exerted contrasting effects on fungal diversity between organs. In leaves, nitrogen deposition significantly increased α-diversity indices, particularly under moderate and severe drought. This result aligns with studies showing that moderate nutrient enrichment can enhance microbial diversity by increasing resource heterogeneity or alleviating nutrient limitation [45]. However, in roots, α-diversity declined under nitrogen addition combined with reduced soil moisture, suggesting intensified competitive interactions or altered plant carbon allocation under nutrient-rich conditions [46]. Nitrogen enrichment has been shown to reduce fungal diversity in some soil systems by favoring fast-growing or competitive taxa [47]. Therefore, nitrogen effects appear context-dependent and strongly mediated by water availability and host physiology.

Beta diversity analysis further revealed significant separation among treatments, particularly under combined severe drought and nitrogen addition. These patterns were further supported by PERMANOVA analysis, which confirmed significant shifts in fungal communities under different treatment combinations. Interaction effects between water deficit and nitrogen input are increasingly recognized as critical drivers of microbial community reassembly under global change scenarios [48]. Changes in host physiological status, including carbon assimilation rates and nutrient partitioning, may indirectly reshape associated fungal assemblages [49]. The robust NMDS stress values support the reliability of these compositional shifts.

Collectively, these findings suggest that global change drivers influence not only species richness but also dominance patterns, niche structure, and community stability of endophytic fungi [50]. The differential responses between leaves and roots emphasize the necessity of incorporating organ-level resolution when evaluating microbial responses to environmental change [51]. Moreover, the observed transition from dominance-driven to more even communities under stress may enhance functional redundancy and ecosystem resilience, a phenomenon increasingly discussed in the context of microbial-mediated plant adaptation to climate change [52,53].

It should be noted that this study focused on culturable endophytic fungi, which may represent only a subset of the total fungal community. Many taxa are not readily culturable under standard conditions, potentially leading to an underestimation of diversity. High-throughput sequencing approaches can provide a more comprehensive view of community composition, whereas culture-based methods remain essential for functional studies [54]. Future work integrating both approaches would improve our understanding of endophytic fungal communities.

### 4.2. Functional Differentiation of DSE Strains and Their Regulatory Mechanisms Under Nitrogen Deposition and Drought

The four DSE strains exhibited clear functional differentiation in colonization ability, growth promotion, physiological regulation, and nutrient acquisition, indicating strain-specific ecological strategies under combined drought and nitrogen deposition stress [55,56]. Although Tm showed the highest colonization rate, its growth-promoting effect was not proportionally greater than that of Av, suggesting that colonization intensity alone does not determine functional outcome. Previous studies have emphasized that DSE–host interactions range from mutualistic to conditionally parasitic, depending on fungal genotype, host status, and environmental context [57,58,59]. Therefore, functional performance likely reflects metabolic compatibility and resource exchange efficiency rather than mere colonization extent.

In terms of biomass accumulation, Av significantly enhanced seedling growth, whereas Cg reduced biomass, highlighting the dualistic nature of DSE symbiosis [60]. Such contrasting responses are commonly observed in DSE–plant associations, where some strains promote nutrient acquisition and stress tolerance, while others impose carbon costs exceeding benefits [61]. The reduction in root-to-shoot ratio observed under Av and Tm inoculation may reflect altered carbon allocation patterns [62], potentially optimizing aboveground photosynthetic investment under stress conditions. Adjustments in biomass allocation are recognized as key adaptive strategies enabling plants to cope with water limitation [63,64], and DSE-mediated modulation of allocation may therefore enhance host performance under drought.

DSE inoculation significantly influenced photosynthetic parameters, particularly net photosynthetic rate (*P*_n_) and water-use efficiency (WUE). Av, Pc, and Tm markedly increased *P*_n_, while Cg and Tm significantly enhanced WUE. Improvements in photosynthetic performance following DSE colonization have been reported in several host species and are often attributed to enhanced nutrient supply, hormonal modulation, or improved hydraulic conductivity [65,66,67]. The increase in WUE, especially under Tm inoculation, suggests that certain DSE strains may confer drought resilience by optimizing stomatal regulation and reducing transpirational water loss. Such physiological adjustments are critical under combined drought and nitrogen stress, where carbon assimilation and water conservation must be tightly balanced.

Interestingly, classical antioxidant enzyme activities (e.g., SOD and CAT) were only marginally affected, whereas osmotic and membrane damage indicators exhibited clearer responses. The significant reduction in proline content across inoculated treatments suggests alleviation of osmotic stress, as proline accumulation is typically associated with drought-induced stress responses [68,69,70]. Similarly, the decrease in MDA under Cg inoculation indicates reduced lipid peroxidation and membrane damage. These results imply that DSE-mediated stress mitigation may rely more on metabolic reprogramming and osmotic adjustment than on activation of enzymatic antioxidant systems, consistent with findings that endophytes can modulate host metabolic pathways to enhance stress tolerance [65,71,72].

Nutrient acquisition patterns further demonstrated strong strain-specific effects. Pc markedly enhanced P, K, and B contents, suggesting a nutrient-promoting functional type. DSE fungi are known to improve mineral nutrient acquisition by producing organic acids, phosphatases, or siderophores that mobilize otherwise unavailable nutrients in soil [73,74]. The universal increase in leaf Zn content across treatments is particularly noteworthy, as Zn plays critical roles in enzymatic activity and stress resistance [75]. Enhanced micronutrient acquisition may therefore contribute to improved photosynthetic and physiological performance observed under certain DSE inoculations.

Hierarchical clustering of standardized traits revealed coordinated functional modules, with growth, photosynthesis, and nutrient variables forming a positive association cluster, while stress indicators such as proline and MDA formed an opposing module. This pattern suggests functional trade-offs and coordinated regulation among physiological pathways. Av and Pc clustered together, representing a “growth–nutrient facilitation” strategy, whereas Tm formed a distinct cluster characterized by improved WUE and carbohydrate accumulation. Cg grouped closer to the CK, indicating comparatively weaker or context-dependent effects. Such differentiation highlights ecological niche specialization among DSE strains and suggests potential functional complementarity within natural endophytic communities [58,59].

Overall, these results demonstrate that DSE fungi play multifaceted roles in modulating host physiology under combined global change stressors [76]. Rather than functioning as uniform mutualists, DSE strains exhibit diverse ecological strategies that influence plant growth, resource allocation, and stress tolerance in distinct ways [77]. In parallel, drought and nitrogen deposition differentially reshaped endophytic fungal communities in leaves and roots, with more pronounced compositional shifts under combined stress conditions. From an applied perspective, strains such as Av and Pc, which enhanced plant growth and nutrient acquisition, may serve as potential bioinoculants for afforestation or ecological restoration in stress-prone environments, while the variability among strains highlights the importance of careful selection in practical applications. Together, these findings highlight the importance of linking community-level responses with strain-specific functional differentiation when evaluating plant–microbe interactions under global change scenarios.

## 5. Conclusions

This study demonstrates that drought and nitrogen deposition significantly reshape the community composition and structure of culturable endophytic fungi in *Q. dentata*, with clear organ-specific responses. Root-associated fungal communities showed greater compositional stability, whereas leaf communities were more sensitive to environmental variation. The combined effects of drought and nitrogen deposition led to pronounced shifts in community structure, highlighting strong interaction effects under global change conditions. DSE strains exhibited clear functional differentiation in colonization ability, growth promotion, photosynthetic regulation, and nutrient acquisition, reflecting diverse ecological strategies. These strains influenced host performance primarily through coordinated regulation of photosynthesis, biomass allocation, and nutrient uptake rather than through strong activation of antioxidant systems.

Future studies should integrate culture-dependent approaches with high-throughput sequencing to better capture the full diversity of endophytic fungi. It will also be important to investigate the functional roles and interactions among multiple DSE strains under varying drought and nitrogen regimes. Their long-term effects on plant performance, nutrient cycling, and forest ecosystem resilience under global change should also be assessed.

## Figures and Tables

**Table 1:** Root colonization rate (%) of *Quercus dentata* by different DSE strains.

Treatment	CK	Cg	Av	Pc	Tm
Colonization rate (%)	0	67.5	40	57.5	82.5

Note: CK represents the non-inoculated control treatment. Cg, Av, Pc, and Tm represent inoculation with *Chaetomium globosum*, *Acrocalymma vagum*, *Paraphoma chrysanthemicola*, and *Tetracladium maxilliforme*, respectively.

## Data Availability

The original contributions presented in this study are included in the article. Further inquiries can be directed to the corresponding author.
